# Germ cell specific overactivation of WNT/βcatenin signalling has no effect on folliculogenesis but causes fertility defects due to abnormal foetal development

**DOI:** 10.1038/srep27273

**Published:** 2016-06-06

**Authors:** Manish Kumar, Nicole J. Camlin, Janet E. Holt, Jose M. Teixeira, Eileen A. McLaughlin, Pradeep S. Tanwar

**Affiliations:** 1 Gynaecology Oncology Group, School of Biomedical Sciences and Pharmacy, University of Newcastle, Callaghan, New South Wales, Australia; 2 Reproductive Science Group, School of Environmental and Life Sciences, University of Newcastle, Callaghan, New South Wales, Australia; 3 Department of Obstetrics, Gynaecology and Reproductive Biology, College of Human Medicine, Michigan State University, 333 Bostwick Ave NE, 4018A, Grand Rapids, MI, USA; 4 Department of Women’s Health, Spectrum Health Systems, Grand Rapids, MI, USA

## Abstract

All the major components of the WNT signalling pathway are expressed in female germ cells and embryos. However, their functional relevance in oocyte biology is currently unclear. We examined ovaries collected from TCFGFP mice, a well-known Wnt reporter mouse model, and found dynamic changes in the Wnt/βcatenin signalling activity during different stages of oocyte development and maturation. To understand the functional importance of Wnt signalling in oocytes, we developed a mouse model with the germ cell-specific constitutive activation of βcatenin using cre recombinase driven by the *DEAD (Asp-Glu-Ala-Asp) box protein 4* (*Ddx4*) gene promoter. Histopathological and functional analysis of ovaries from these mutant mice (Ctnnb1^ex3^cko) showed no defects in ovarian functions, oocytes, ovulation and early embryonic development. However, breeding of the Ctnnb1^ex3^cko female mice with males of known fertility never resulted in birth of mutant pups. Examination of uteri from time pregnant mutant females revealed defects in ectoderm differentiation leading to abnormal foetal development and premature death. Collectively, our work has established the role of active WNT/βcatenin signalling in oocyte biology and foetal development, and provides novel insights into the possible mechanisms of complications in human pregnancy such as repeated spontaneous abortion, sudden intrauterine unexpected *foetal death syndrome* and stillbirth.

WNT signalling is involved in various developmental processes such as cell proliferation and differentiation^1^. βcatenin is an important mediator of the WNT pathway. In the absence of WNT ligands, the Adenomatous Polyposis Coli (APC) complex binds to βcatenin and causes its phosphorylation at highly conserved Ser/Thr residues^1^. This phosphorylated form of βcatenin is then recognised by the E3 ubiquitin ligase complex and degraded by the proteasome. In the presence of WNT ligands, the APC complex is no longer able to target βcatenin, leading to the stabilization and subsequent nuclear localization of βcatenin^1^. In the nucleus, βcatenin interacts with various factors, including the members of the TCF/LEF family to activate the transcription of targeted genes^2^.

We and others have established the significance of WNT signalling in gonadal functions and reproductive tract cancers^3^,^4^,^5^,^6^,^7^. Overexpression of the *Wnt4* gene, a well-known WNT ligand, causes suppression of steroidogenesis and abnormal testis development in both humans and mice^8^. In contrast, loss of the *Wnt4* gene leads to female-to-male sex reversal in mice^9^. In male mice, overactivation of WNT signalling by the conditional deletion of exon3 of the β*catenin* gene (*Ctnnb1*) in Sertoli cells of testis causes disorganization of seminiferous tubules, premature germ cell loss, infertility and stromal tumours^5^,^7^. Loss of *Apc*, a negative regulator of βcatenin, in stromal cells induces an epithelial-mesenchymal transition-like state in Sertoli cells leading to defects in testicular junctional complexes and infertility^4^. Similar genetic alterations in the *CTNNB1* and *APC* genes are also present in human Sertoli cell tumour patients^10^,^11^, suggesting that deregulated WNT signalling is involved in the pathogenesis of testicular cancer.

WNT/βcatenin signalling has been shown to be involved in ovarian development and diseases^12^,^13^. Many members of the WNT pathway including ligands, receptors, co-receptors and negative regulators are expressed in the mouse ovary, oocyte and embryos^14^. Somatic cell-specific loss of the *Wnt4* gene in the mouse ovary using Anti Müllerian hormone receptor 2 cre (Amhr2cre) causes abnormalities in follicular development resulting in premature ovarian failure and subfertility^15^,^16^. Similar to WNT4, Frizzled4, a known receptor for WNT ligands, is expressed in the mouse ovary and its loss causes impairment of corpus lutea development and infertility without affecting follicular formation and functions^17^. Stabilization of βcatenin in postnatal granulosa cells increases follicle stimulating hormone (FSH)-induced growth of ovarian follicles but suppresses luteinising hormone (LH)-induced oocyte maturation, ovulation and luteinisation^18^. In prenatal granulosa cells, constitutive activation of βcatenin causes defects in the differentiation of granulosa cells, leading to the development of stromal tumours^13^. Activation of PI3K signalling by deleting *Pten* in these mice results in the development of a highly aggressive and metastatic form of ovarian stromal tumours^19^. Collectively, these findings highlight the importance of the WNT pathway in stromal cells of the ovary.

Although much is known about the functions of WNT signalling in the stromal cells of the ovary, limited information is available regarding its involvement in ovarian germ cell biology. To determine the significance of WNT signalling during different stages of oogenesis, we monitored real time changes in WNT signalling activity in the mouse ovary using a well-known WNT reporter mouse model. We found dynamic changes in the levels of WNT activity during different stages of oocyte development and maturation suggesting a requirement for this pathway in follicle/oocyte functions. To further understand the requirement of WNT signalling in oocyte biology, we developed a mouse model with overactive βcatenin in female germ cells and showed that abnormal WNT/βcatenin signalling leads to defects in female fertility.

## Results

### Activation of WNT signalling during different stages of follicular development

**Figure 1:**
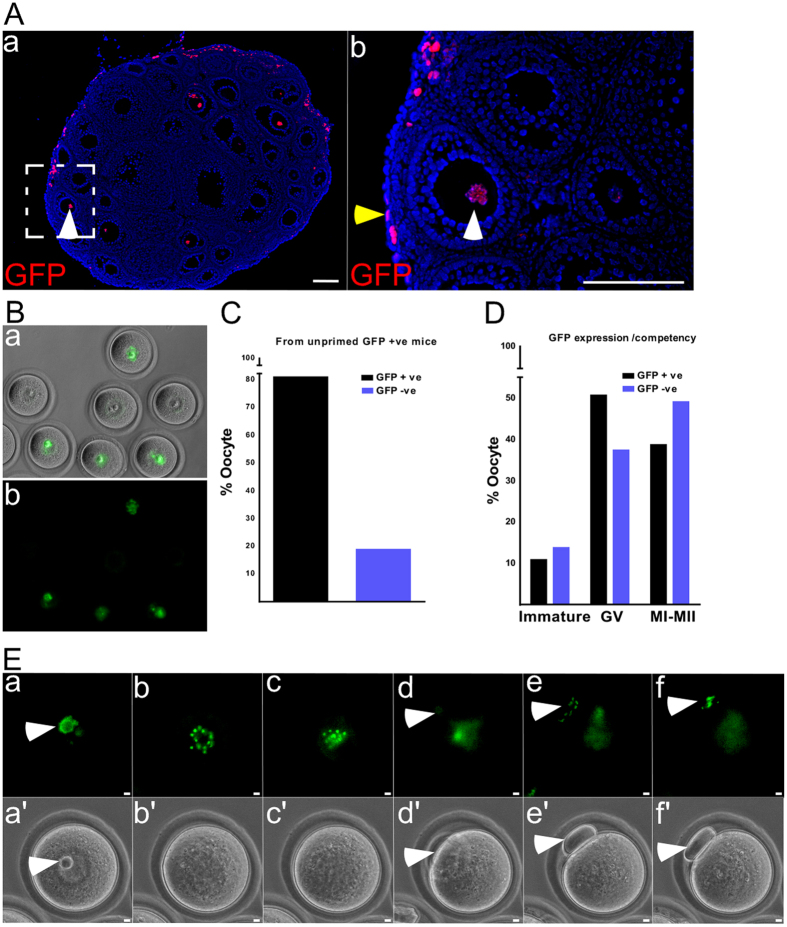
Active WNT signalling during follicular development in mouse ovary. Nuclear GFP expression (white arrowhead), indicative of active Wnt signalling, is present in oocytes of TCFGFP ovaries (**A**a,b). GFP expression was also observed in ovarian surface epithelium (**A**b; yellow arrowhead). Brightfield and GFP images of oocytes collected from ovaries of unprimed TCFGFP mice (**B**a,b). Both GFP positive and negative oocytes were present. Graphical presentation of percentage of GFP positive oocytes collected from non-cycling TCFGFP ovaries (**C**; N = 26). No significant difference between GFP positive and negative oocytes were observed post *in vitro* maturation for the different oocyte stages that were, immature oocytes (with intact nuclear membrane), Germinal Vesicle (GV; competent to undergo nuclear membrane breakdown), MI-MII (full meiotic competence-extrudes polar body *in vitro*) (**D**). Sequential imaging of a TCFGFP oocyte undergoing *in vitro* maturation showed higher GFP expression in the extruding polar body compared to the rest of the oocyte. Bars: 100 μm (**A**); 10 μm (**E**).

To determine the physiological activity of WNT signalling during follicular development, we examined ovaries from a well-characterized Wnt reporter mouse model, TCFGFP. In this model, nuclear GFP is only present in cells with active WNT signalling^20^. Examination of ovaries from pre-pubertal TCFGFP mice revealed GFP expression in the nucleus of oocytes (Fig. 1A; N = 3). Both GFP positive and negative oocytes were present in TCFGFP ovaries. Consistent with previous reports showing active WNT signalling in the ovarian surface epithelium (OSE) and stromal cells^21^, GFP expression was also observed in OSE and some stromal cells of ovaries collected from TCFGFP mice (Fig. 1A; N = 3). Next, we collected oocytes from unprimed TCFGFP mice (N = 3) and found that 81% (N = 21/26) of oocytes were positive for GFP expression (Fig. 1B,C). To study dynamic changes in WNT signalling during oocyte maturation, oocytes collected from primed TCFGFP ovaries were subjected to undergo *in vitro* maturation. We observed no difference in the meiotic competency of GFP positive and negative oocytes during different stages of oocyte maturation (Fig. 1D). This suggests that active WNT signalling is not a predictor of oocyte meiotic competency. Time lapse imaging of *in vitro* maturation of oocytes revealed changes in GFP expression during different stages (Fig. 1E). High GFP expression was also found in the extruding polar body (Fig. 1Ee–f) indicating a role for WNT signalling in asymmetric division. Collectively, these results showed that dynamic changes in WNT signalling activity occur during several stages of oocyte development and maturation.

### Conditional activation of WNT/βcatenin signalling in ovarian germ cells

**Figure 2:**
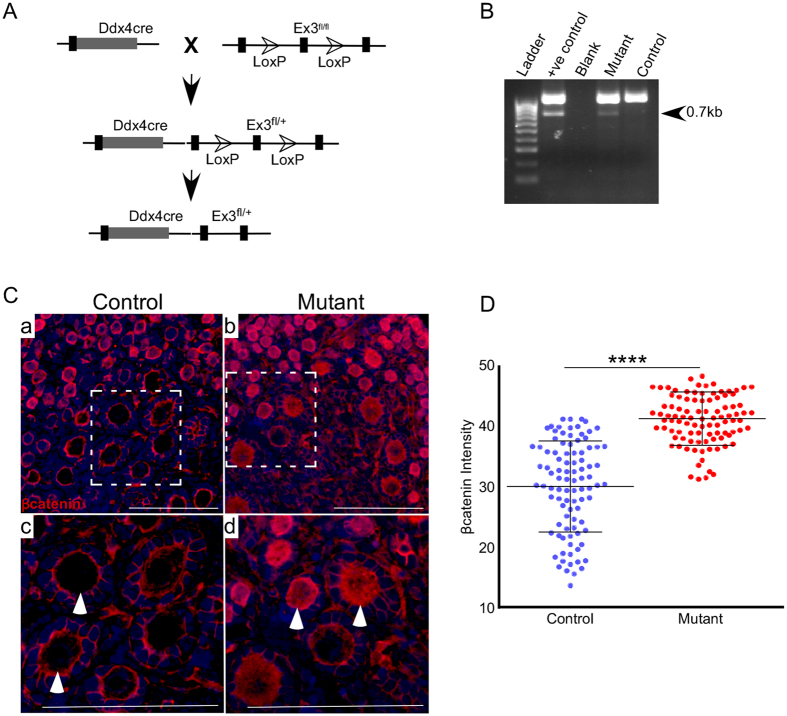
Overactivation of Wnt signalling in oocytes does not affect oogenesis. (**A**) Schematic representation of mouse model with germ cell-specific overactivation of Wnt signalling. (**B**) PCR based detection of the mutant form (arrow) of the *βcatenin* gene from DNA isolated from ovaries of control and Ctnnb1^ex3^cko mice. Presence of 700 bp band with DNA from ovaries of mutant animals shows successful recombination. (**C**) Increased expression of βcatenin in oocytes of mutant mice (arrowheads) as compared to control (arrowheads) indicating of overactive Wnt signalling in germ cells of mutant ovaries. No change in βcatenin expression was observed in ovarian somatic cells in both control and mutant animals. (**D**) Quantification of βcatenin expression in oocytes of control and mutant ovaries showing a significant increase in intensity of βcatenin staining in oocytes of mutant ovaries. Nuclei are marked blue by DAPI. Bars: 100 μm.

To further investigate the role of active WNT signalling in oocyte development, we developed a mouse model with germ cell specific overactivation of WNT/βcatenin signalling using DEAD (Asp-Glu-Ala-Asp) box protein 4 cre (Ddx4cre). In our previous study, we have shown that *Ddx4cre* causes faithful recombination in ovarian germ cells beginning from embryonic day 15^22^. We crossed Ddx4cre mice with Ctnnb1^tm1Mmt^ mice to generate mice with deletion of exon 3 of the *βcatenin* gene (Fig. 2A; Ctnnb1^ex3^cko). Exon 3 of the β*catenin* gene harbours phosphorylation sites targeted by the APC destruction complex leading to abnormal accumulation of βcatenin in the cytoplasm that subsequently translocates to the nucleus leading to the transcription of targeted genes^23^. Using PCR, we confirmed that Ddx4cre specifically causes recombination of Ctnnb1^tm1Mmt^ flox allele in mutant mice (Fig. 2B). Examination of βcatenin protein expression showed a significant increase in cytoplasmic and nuclear accumulation of βcatenin in the oocytes of mutant ovaries compared to controls (Fig. 2C,D; N = 3). No change in localization of βcatenin was observed in both control and mutant ovarian somatic cells (Fig. 2C). To further confirm the specificity of Ddx4cre, we developed another mouse model (Ctnnb1^ex3/lacZ^cko) and found presence of LacZ/βgal expression only in germ cells of mutant ovaries (SFig. 1A–P; N = 3/3). Colocalization of βgal and βcatenin revealed cytoplasmic/nuclear accumulation of βcatenin only in the βgal-positive oocytes of the mutant ovaries (SFig. 2A–D; N = 3/3). Co-immunostaining of βgal with a germ cell marker^4^ (GCNA: Germ Cell Nuclear Antigen) showed that βgal expression in the mutant ovaries is limited to the GCNA-positive cells (SFig. 2E–H; N = 3/3). In summary, these results demonstrated that Ddx4cre mediated deletion of exon3 of β*catenin* gene causes a greater accumulation of βcatenin specifically in germ cells of the ovary.

### Oocyte specific constitutive activation of WNT/βcatenin signalling causes subfertility but has no effect on folliculogenesis and early embryonic development

To study the effects of overactivation of WNT/βcatenin signalling, control (N = 14) and mutant females (Ctnnb1^ex3^cko; N = 10) were mated with the wild type and Ctnnb1^tm1Mmt^ males of known fertility. Copulatory plugs were observed in both control and mutant females, suggestive of normal mating behaviour in these mice. Control females bred normally and produced 5.09 ± 1.97 pups per litter during four months of breeding period, whereas, mating of mutant females with Ctnnb1^tm1Mmt^ males produced no litters (Table 1). Fewer pups were born from the mating of mutant females with wild type males (Table 1). Genotyping of pups resulting from this mating showed none of the pups were of the mutant genotype (Ctnnb1^ex3^cko).

**Figure 3:**
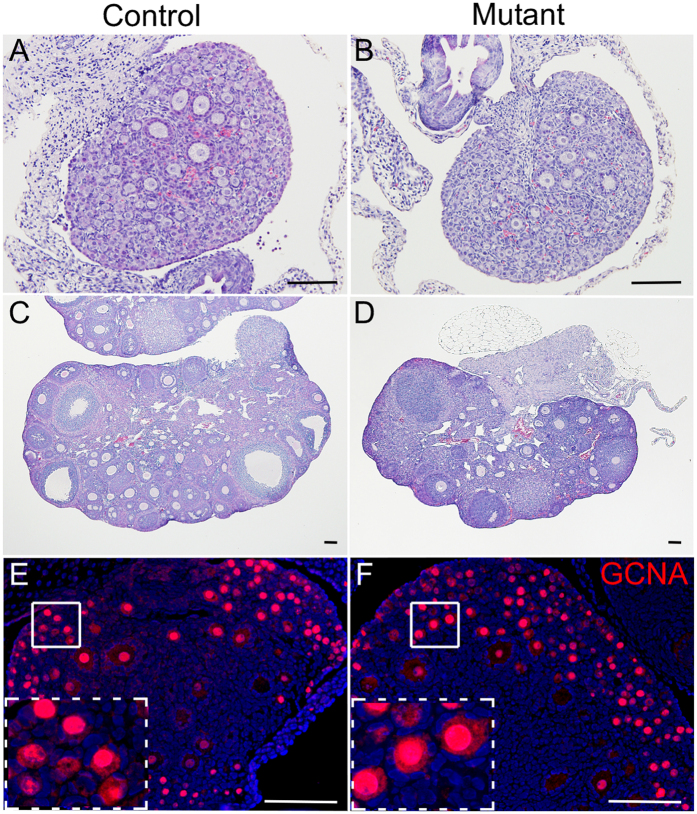
Normal morphology of pre-pubertal and adult ovaries in control and mutant mice. Histology of early postnatal (**A,B**; 3-day-old) and adult (**C**,**D**; 7-weeks-old) ovaries from control and mutant mice showed no obvious differences. GCNA, a marker of germ cells, expression in ovaries of 3-day-old control (**E**) and mutant (**F**) mice. Nuclei are marked blue by DAPI. Bars: 100 μm.

**Figure 4:**
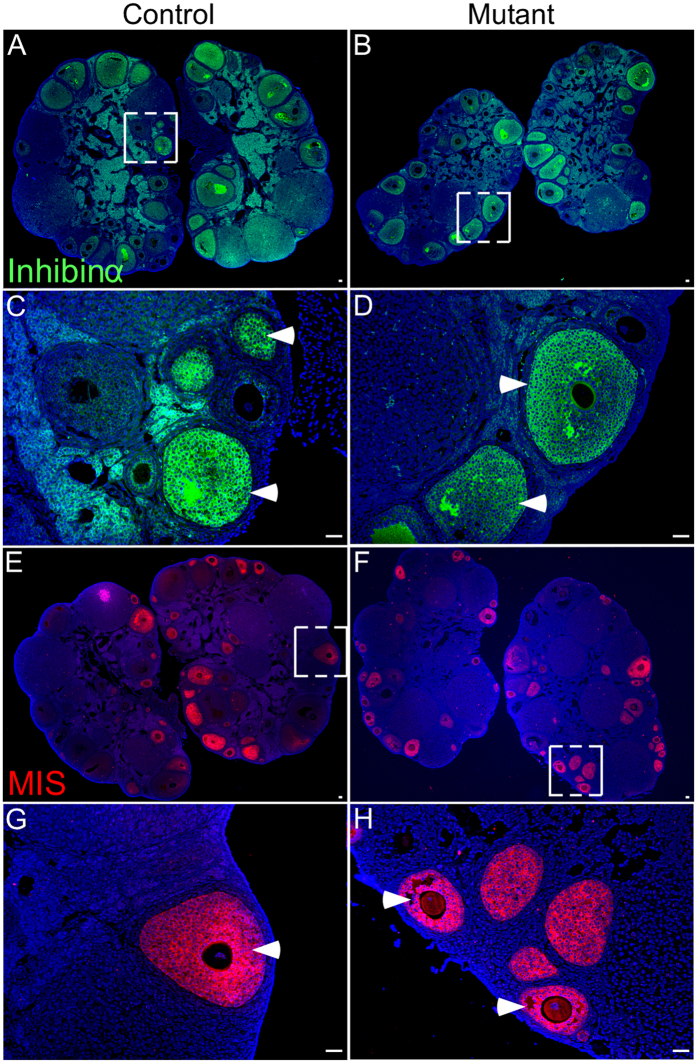
Normal follicular development in ovaries of control and mutant mice. Inhibinα, a maker for antral follicles, expression in control (**A**,**C**) and mutant (**B**,**D**) ovaries. Panel **C** and **D** are high magnification images of boxed areas in panel **A** and **B**, respectively. Expression of Müllerian Inhibiting Substance (MIS), a marker for preantral follicles, was also similar among control (**E**,**G**) and mutant ovaries (**F,H**). Nuclei are marked blue by DAPI. Bars: 100 μm.

Histological examination of pre- and post-pubertal ovaries showed no difference between control and mutant mice (Fig. 3A–D; N = 5/each). Corpora lutea were present in adult control and mutant ovaries suggesting that ovulation was normal in these females (Fig. 3C,D; N = 5/each). To determine if overactive βcatenin affects the germ cells, we performed immunostaining for GCNA and found no obvious difference in GCNA expression in oocytes from ovaries in both groups (Fig. 3E,F; N = 5/each). Examination of inhibinα (Fig. 4A–D; N = 5/each), a marker for antral follicles^12^, and Müllerian Inhibiting Substance/Anti Müllerian hormone (AMH) (Fig. 4E–H; N = 5/each), a marker for developing follicles^12^, expression in ovaries also revealed no difference between control and mutant mice. These findings suggest that germ cell and follicular development is not affected in mutant mice.

**Figure 5:**
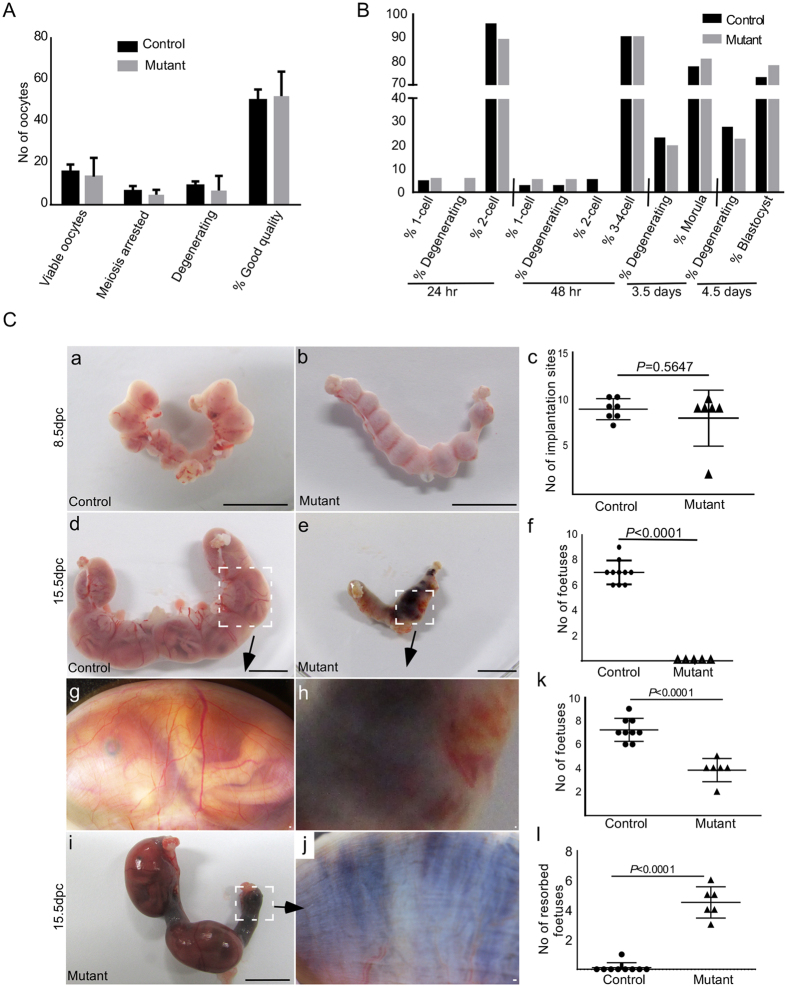
Overactivation of Wnt signalling in oocytes does not affect oocyte maturation but leads to fertility defects due to embryonic death. (**A**) No obvious differences were observed in oocytes (viable, meiosis arrested, degenerating and good quality) that were collected by hormonal priming of control and mutant ovaries. (**B**) No differences were observed among various stages of parthenogenetic development (1-cell, degenerating cells, 2-cells, 3–4-cells, morula and blastocyst) of oocytes collected from control and mutant animals. (**C**a,b) Normal gross morphology of pregnant uteri from control and mutant females at 8.5 dpc. (**C**c) Graph represents no significant difference between the number of implantation sites in control (N = 7) and mutant (N = 6) animals at 8.5 dpc. (**C**d,g) Normal foetal development was observed in 15.5 dpc control females (N = 10). Blackened and shrunken uterus without any viable foetuses collected from a 15.5 dpc mutant female mated with a Ctnnb1^tm1Mmt^ male (**C**e,h; N = 5). (**C**f) Graph depicts significant difference in the number of foetuses at 15.5 dpc between control and mutant females. (**C**i,j) Both live and resorbed foetuses were present in the pregnant uteri of mutant females that were mated with wild type males at 15.5 dpc. (**C**k,l) Total number of foetuses and the number of resorbed foetuses in 15.5 dpc control and mutant females mated with wild type males. Panel Cj is high magnification image of boxed area in panel Ci. Bars: 1 cm (**C**, a,b,d,e and i) and 100 μm (**C,**g–h and j).

Our analysis of TCFGFP mice showed dynamic changes in WNT/βcatenin signalling occurs during different stages of oocyte maturation (Fig. 1E). To determine the effect of overactive WNT signalling on oocyte maturation, we collected oocytes from hormonally primed control and mutant mice (Ctnnb1^ex3^cko; N = 3/each). The number of oocytes collected from mutant ovaries was comparable to controls (Fig. 5A). To evaluate the potential of the mutant oocyte to undergo embryogenesis, we subjected both control and mutant oocytes to parthenogenetic activation (N = 3/each). Both control and mutant oocytes showed comparable potential to undergo parthenogenesis and subsequent embryonic development to blastocyst stage (Fig. 5B). Collectively, these data suggest that the process of oocyte maturation and early embryonic development is not affected by hyperactive WNT signalling.

### Sustained activation of WNT signalling in oocytes leads to abnormal development of ectoderm and foetal loss

**Figure 6:**
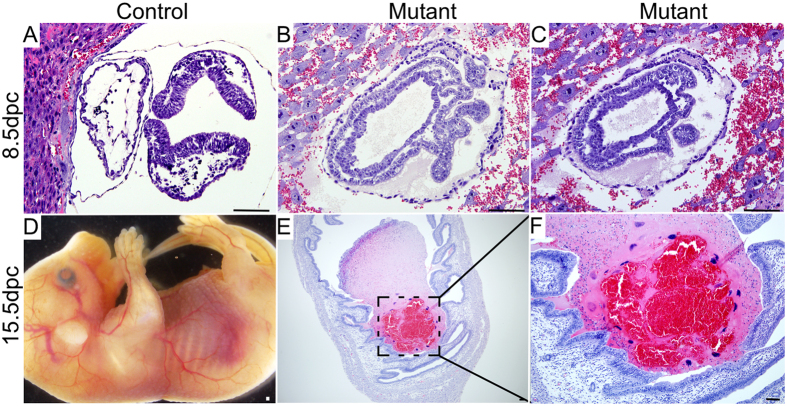
Defective embryonic development in mutant mice. Representative images of 8.5 dpc embryos from control (**A**) and mutant (**B**,**C**) mice. (**D**) Gross image of 15.5 dpc foetus of control mice. (**E**,**F**) H and E image showing the site of resorbed fetus in 15.5 dpc pregnant uteri of mutant mice. Bars: 100 μm.

To determine whether abnormalities during the later stages of embryonic development cause defects in fertility of the mutant mice, we time mated control and mutant females with Ctnnb1^tm1Mmt^ males of proven fertility. Control and mutant uteri from time pregnant females were collected during different stages of foetal development. Examination of 8.5 day post coitum (dpc) pregnant uteri showed no differences in the number or apparent size of implantation sites between control (N = 7) and mutant (N = 6) mice (Fig. 5Ca,b). Histological analysis of implantation sites confirmed normal implantation of embryos in both groups of mice (Fig. 6A–C). Analysis of 10.5dpc gravid uteri also showed no difference in the number of implantation sites between control and mutant mice (SFig. 3A–C). However, closer examination of implantation sites revealed reduced uterine vascular network and decrease in weight of implantation sites in mutants compared to controls (SFig. 3D–I). Comparison of control and mutant gravid uteri showed that the size of 13.5 dpc mutant implantation sites is smaller than the size of 10.5 dpc control implantation sites (SFig. 4), suggesting defective embryonic development in mutant females. Histological examination of 10.5 dpc pregnant uteri indicated normal embryonic development in control females (SFig. 3G). However, embryonic development is compromised in 10.5 dpc mutant pregnant uteri (SFig. 3H).

Next, we examined uteri from pregnant mutant dams at 15.5 dpc and observed black discoloured uteri, indicative of dead/resorbed foetuses (Fig. 5Ce,h; N = 5/5). Normal foetal development was observed in control females (Fig. 5Cd,g; N = 10/10). Histological examination of 15.5 dpc pregnant uteri from the mutant females revealed disorganised pink/red necrotic mass at the site of implantation, which further confirmed abnormalities during foetal development (Fig. 6D–F). To further examine the fertility of mutant mice we time mated the mutant females (N = 6) with wild type males of known fertility. We observed reduction in average pup number per litter (3.8 ± 1.13; Table 1). None of the pups (N = 38) from 10 litters were of the mutant genotype. To confirm if foetal mortality is the reason for reduced litter size, we collected uteri from 15.5 dpc pregnant mutant females and found a significant number of dead foetuses (4.5 ± 1/litter) in mutant mice (Fig. 5Ci–l).

**Figure 7:**
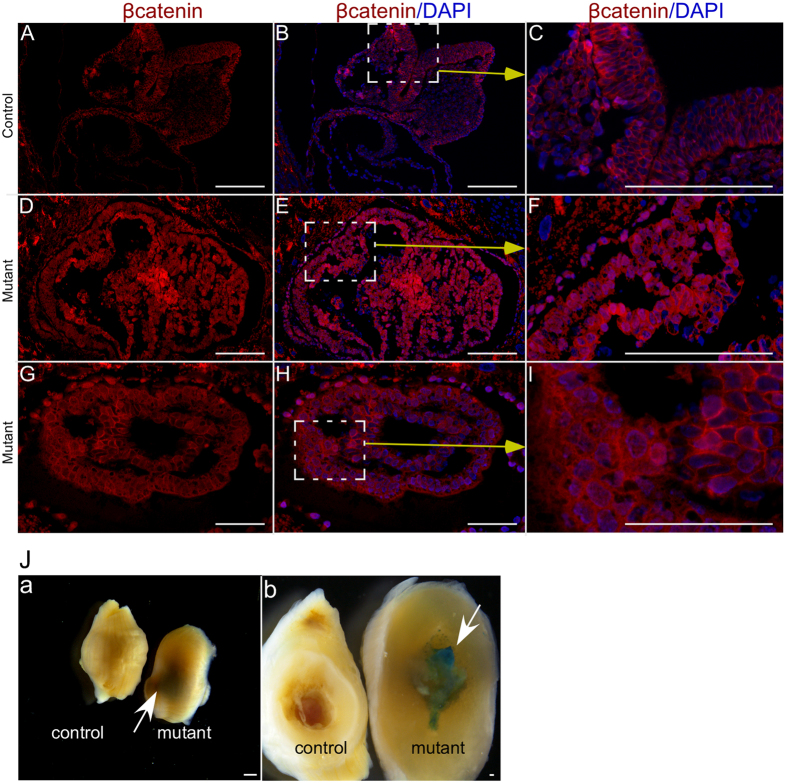
Embryonic lethality in mutant females is associated with overactivation of Wnt signalling in embryos. (**A–C**) 8.5 dpc embryos from control females showed membranous expression of βcatenin. (**D–I**) Cytoplasmic and nuclear accumulation of βcatenin, indicative of overactive Wnt signalling, in 8.5 dpc embryos from mutant females. (**J**a,b) lacZ expression was present only in embryos and not in other cells of uterus collected from 8.5 dpc Ctnnb1^ex3/lacZ^cko females. No lacZ expression was observed in control Ctnnb1^ex3/+^;ROSA26^flGFP-NLS-lacZ/+^ mice. White arrow marks lacZ positive embryo. Nuclei are marked blue by DAPI. Bars: 100 μm.

**Figure 8:**
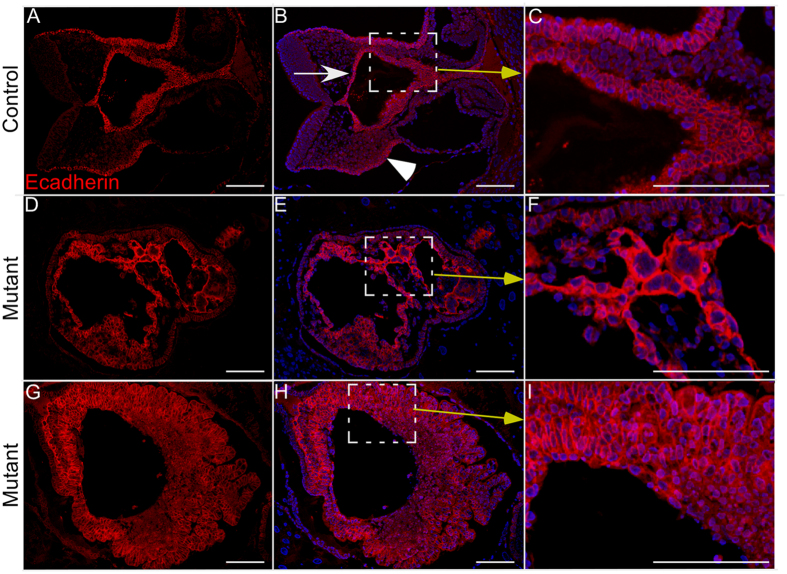
Overactivation of Wnt signalling lead to defective germ layer differentiation in the embryos of mutant mice. **(A–C**) Ecadherin expression present in the endoderm (marked by white arrow) and lateral thin ectoderm (marked by white arrowhead) of 8.5 dpc mouse embryo from control females. (**D–I**) No specific Ecadherin expression pattern was observed as most of the cells were positive in embryos of mutant females indicating defective embryonic germ layer differentiation during early stages of embryonic development. Nuclei are marked blue by DAPI. Bars: 100 μm.

**Figure 9:**
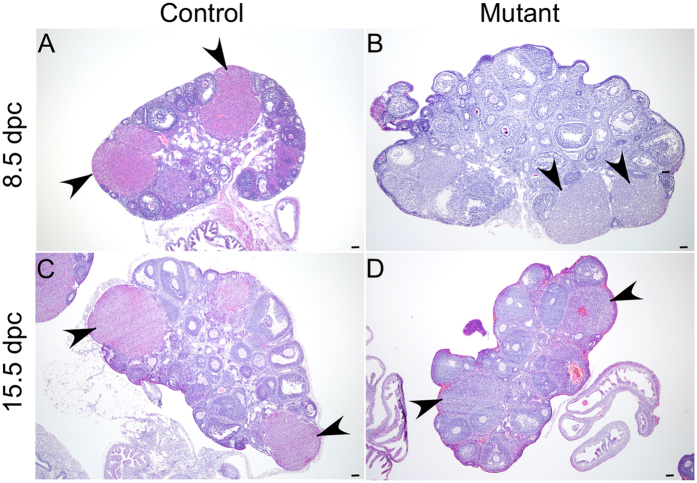
Normal corpus luteum development in control and mutant females. H and E stained sections of control (**A**) and mutant (**B**) ovaries showing normal development of corpus luteum of 8.5 dpc pregnant mice. Normal development of corpora lutea continued at 15.5 dpc in control (**C**) and mutant ovaries (**D**). Arrowheads marks corpus luteum. Bars: 100 μm.

To investigate the reasons for foetal lethality in mutant females, we performed histological examination of 8.5 dpc embryos and found a disorganised development of all three germ layers in mutants compared to controls (Fig. 6A–C). We selected the 8.5 dpc time point because at this stage there was no gross difference in the number or size of implantation sites (Fig. 5Ca–c). Analysis of βcatenin expression in embryos collected from 8.5 dpc time mated control females depicted only membranous expression of βcatenin in controls (Fig. 7A–C). However, both cytoplasmic and nuclear accumulation of βcatenin was seen in embryos collected from mutant mice (Fig. 7D–I). LacZ expression only in the mutant embryos from the Ctnnb1^ex3/lacZ^cko dams confirmed recombination in embryonic tissues (Fig. 7Ja,b). Next, we examined the expression of Ecadherin, a marker for the endodermal layer of the gut and the surface ectoderm of 8.5 dpc embryos^24^, in control and mutant mice (Fig. 8A–I). Compared to controls (Fig. 8A–C), abnormally increased Ecadherin expression was detected in mutant embryos (Fig. 8D–I). A similar expression pattern for βcatenin and Ecadherin was also observed in 10.5dpc mutant embryos (SFigs 5 and 6). This suggests that the embryonic germ layers are not formed normally in mutant embryos. To rule out the possible loss of progesterone to the foetal death phenotype, we analysed control and mutant ovaries from 8.5 and 15.5 dpc pregnant females and confirmed the presence of corpora lutea in ovaries from both groups (Fig. 9A–D). In summary, these findings have demonstrated that constitutive activation of WNT/βcatenin in oocytes causes defects in the development of embryonic germ layers, leading to infertility/subfertility due to foetal death.

## Discussion

WNT signalling plays a major role in organogenesis and oncogenesis of the reproductive tract^3^,^13^,^25^,^26^. We have previously shown that balanced WNT signalling is essential for the proper development of reproductive tract organs and fertility^3^,^4^. Deregulated WNT signalling causes defects in gonadal cell proliferation and differentiation resulting in the genesis of ovarian and testicular cancers^4^,^26^,^27^. Importantly, 71% of Sertoli cell tumour patients present with activating mutations in the *Ctnnb1* gene and show cytoplasmic/nuclear accumulation of this protein^10^. Similarly, 16–38% of human ovarian endometrioid adenocarcinoma patients, a subtype of ovarian epithelial cancer, harbour activating mutations in the β*catenin* gene^28^. In mouse models, activation of WNT/βcatenin signalling by the deletion of exon 3 of β*catenin* or truncation of *Apc* results in the development of similar ovarian and testicular tumours^12^,^26^,^28^, providing strong evidence for the involvement of this pathway in the pathogenesis of these cancers. Whether dysregulated WNT signalling also contributes to the formation of ovarian germ cell tumours is currently unknown. In this study, we have shown that sustained activation of βcatenin in ovarian germ cells is unable to initiate tumorigenic growth of these cells, suggesting limited involvement of this pathway in ovarian germ cell tumours.

In mice, primordial germ cells (PGCs) differentiate from the epiblast at ~7 dpc and migrate through the hindgut and dorsal mesentery to reach the genital ridges by 10.5 dpc^29^. During early development, these cells undergo massive expansion and their number increases from ~45 at 7.5 dpc to ~25000 at 13 dpc^29^. In the gonads, PGCs acquire male or female fate under the influence of sex-specific signalling pathways. Any disruption to these signalling molecules leads to aberrations in proliferation and/or differentiation of these cells^29^. In mice, loss of *Rspondin 1*, an agonist of WNT signalling, leads to impairment of germ cell proliferation and meiosis^30^. Constitutive activation of WNT signalling by stabilization of βcatenin also causes germ cell deficiency through decreasing primordial germ cell proliferation at the G1/S phase of the cell cycle^31^. These findings suggest that balanced WNT signalling in germ cells is essential for their proliferation and their commitment to meiosis. Surprisingly, in this study, we found no adverse effects from the overactivation of WNT/βcatenin signalling in female germ cells on oocyte development and maturation, ovulation and normal ovarian functions (Figs 3 and 4). The use of a different gene promoter driving cre recombinase expression might explain differences in the phenotype between our study and others^30^,^31^. Both of the previous studies have used *Tissue Non-specific Alkaline phosphatase cre* (*TNAPcre*), which is expressed in pre-meiotic PGCs from 9.5 dpc and also in other organs including the placenta and the intestine^32^. In comparison, *Ddx4cre* is known to induce recombination in meiotic germ cells of the ovary from 15 dpc onwards^33^. Collectively, these findings suggest differential requirements of WNT/βcatenin signalling during different stages of germ cell development.

The follicles are the functional units of the ovary that provide a nourishing environment for normal oocyte development^29^. In each oestrous cycle, some follicles are recruited from the primordial follicle reserve to grow under the influence of gonadotropins and only a few selected ones are ovulated in mice^29^. There are no specific markers to predict which follicles will be recruited to grow and ovulate or undergo atresia. A recent study showed that high WNT signalling activity marks non-ovulatory follicles in mouse ovary^34^. Using TOPGAL mice, a β-galactosidase (lacZ) based WNT signalling reporter mouse model, these authors showed that lacZ staining is mainly present in the oocytes of the post-pubertal ovaries. Both lacZ-positive and -negative follicles are present in TOPGAL ovaries^34^. However, only lacZ-negative oocytes are ovulated and lacZ staining was limited to the atretic oocytes^34^. In this study, we have used TCFGFP mice that are similar to TOPGAL mice except *lacZ* is replaced with the *histone 1 H2bb enhanced green fluorescent fusion protein* gene^20^. In TCFGFP mice, highly specific nuclear GFP is directly detectable in cells where WNT/βcatenin signalling is activated^20^. Using this model, we showed that active WNT signalling is present in both ovulatory and non-ovulatory oocytes (Fig. 1). We observed no differences in meiotic competency of GFP-positive and -negative oocytes (Fig. 1). We believe the differences between our results and the TOPGAL study^34^ can be attributed to the lack of sensitivity of the standard lacZ staining procedure. For example, the traditional method of lacZ staining using X-gal with potassium ferri- and ferro-cyanide (FeCN), which is also used in the TOPGAL study^34^, showed no or very weak lacZ expression in the embryos collected from BAT-Gal Wnt reporter mice, suggesting lack of WNT signalling activity during early embryonic development^35^. However, combination of Salmon-gal with TNBT (5-bromo-4-chloro-3-indoxyl phosphate) or NBT (4-nitro blue tetrazolium chloride) revealed robust lacZ expression in the same stage BAT-Gal embryos^35^.

In summary, we have shown that the physiological activation of WNT/βcatenin signalling occurs during different stages of oocyte development and maturation. Sustained activation of this signalling pathway in female germ cells has no effect on oocyte biology and function, but leads to defects during foetal development.

## Materials and Methods

### Mouse breeding and husbandry

Mice used in this study were housed under standard conditions at the University of Newcastle animal facility. All animals were maintained on C57BL/6;129SvEv mixed genetic background. Animal care and experimental procedures were conducted in accordance with the guidelines of the Animal Care and Ethics Committee of the University of Newcastle, and conformed to the New South Wales Animal Research Act, New South Wales Animal Research Regulation, and the Australian code for the care and use of animals for scientific purposes. All the procedures undertaken on mice were approved by the Animal Care and Ethics Committee of the University of Newcastle. TCF/Lef:H2B/GFP mice used in the present study were obtained from the Jackson lab (ME, USA) and referred as TCFGFP^20^. Ddx4cre (also known as Vasacre) mice^22^ were crossed with Ctnnb1^tm1Mmt ^23 for developing a mouse model (Ctnnb1^ex3^cko) with germ cell specific overactivation of WNT/βcatenin signalling. Ctnnb1^ex3^cko mice were bred with homozygous ROSA26^flGFP-NLS-lacZ^ mice^36^ to obtain Ctnnb1^ex3/lacZ^cko (Ddx4cre;Ctnnb1^ex3/+^;ROSA26^flGFP-NLS-lacZ/+^) mice. DNA isolation and genotyping were performed using REDExtract-N-Amp™ Tissue PCR Kit (Sigma, MO, USA). For recombination PCR, DNA was isolated from whole ovaries of adult control and mutant female mice using the same kit. Sequence of primers used is listed in Table S1.

### Time mating and fertility analysis

For timed pregnancies, control and mutant females were mated with males of known fertility. Females were checked for the presence of copulatory plugs in the morning and the day of plug was considered as 0.5 dpc. Time pregnant females were euthanized at several time points and gravid uteri were collected. Implantation sites were counted and the uteri were fixed in 4% PFA, overnight at 4 °C. For fertility analysis, adult control (Ctnnb1^tm1Mmt^) and mutant (Ctnnb1^ex3^cko) females of reproductive age were paired with Ctnnb1^tm1Mmt^ for four months and the number of litters and pups born per litter were recorded.

### Mouse oocyte collection and maturation

Oocyte collection and maturation was performed as described by us in^37^. The oocytes after *in vitro* maturation (IVM) were scored morphologically for different maturation stages that are, germinal vesicle (GV), germinal vesicle breakdown (GVB), MII determined by the first polar body (PB1) extrusion and oocyte degeneration.

### *In vitro* parthenogenic development

IVM oocytes were activated parthenogenetically using strontium-containing medium as described in our previous study^38^.

### Histology and Immunohistochemistry

Histological and IHC protocols used in this study were adopted from our previous study^39^. 5 μm thick deparaffinised tissue sections were incubated with primary antibodies (Table S2), followed by AlexaFluor secondary antibodies (1:250; Jackson ImmunoResearch Laboratories, PA, USA) for signal detection. Pictures were obtained using an Olympus DP72 microscope or Olympus FV1000 (Olympus, Tokyo, Japan) with the same gain and exposure for tissues from the control and mutant mice. For the assessment of florescence intensity of βcatenin at least 30 oocytes were counted, from three different animals from both control and mutant group, using ImageJ (National Institutes of Health, USA). β-galactosidase staining procedure is described in^40^.

### Statistical Analysis

Statistical significance was calculated by Student *t*-test using GraphPad Prism 6.0 Software with *P* value < 0.05 considered significant. All values are presented as mean  ±  SEM. Each experiment was performed with N ≥ 3 for both control and mutant mice.

## Additional Information

**How to cite this article**: Kumar, M. *et al.* Germ cell specific overactivation of WNT/βcatenin signalling has no effect on folliculogenesis but causes fertility defects due to abnormal foetal development. *Sci. Rep.* **6**, 27273; doi: 10.1038/srep27273 (2016).

## Supplementary Material

Supplementary Information

## Figures and Tables

**Table 1:** *Ctnnb1* ^*ex3*^ *cko* female mice are subfertile.

	Total litters	Total pups	Pups/litter
Ctnnb1^tm1Mmt^ × Ctnnb1^tm1Mmt^	23	121	5.09 ± 1.97
Ctnnb1^tm1Mmt^ × Ctnnb1^ex3^cko	0	0	0
Wild type × Ctnnb1^ex3^cko	10	38	3.8 ± 1.13
