# Supplementary material for: Germ cell specific overactivation of WNT/βcatenin signalling has no effect on folliculogenesis but causes fertility defects due to abnormal foetal development
Source: Sci Rep. 2016 Jun 6;6:27273. doi: 10.1038/srep27273 (PMC4893675; doi:10.1038/srep27273)
Supplement: Supplementary Information [file srep27273-s1.pdf]

Germ cell specific overactivation of WNT/βcatenin signalling has no effect on folliculogenesis but causes fertility defects due to abnormal foetal development

Manish Kumar[1], Nicole J. Camlin[2], Janet E. Holt[2], Jose M. Teixeira[3], Eileen A. McLaughlin[2], Pradeep S. Tanwar[1*]

[1]Gynaecology Oncology Group, School of Biomedical Sciences and Pharmacy; [2]Reproductive Science Group, School of Environmental and Life Sciences, University of Newcastle, Callaghan, New South Wales, Australia [3]Department of Obstetrics, Gynaecology and Reproductive Biology, College of Human Medicine, Michigan State University, 333 Bostwick Ave NE, 4018A, Grand Rapids, MI, USA Department of Women's Health, Spectrum Health Systems, Grand Rapids, MI, USA

Key terms: Wnt, βcatenin, ovary, APC, LEF1, fertility

*Address correspondence to:

Dr Pradeep Tanwar
Australian Research Council Future Fellow
Cancer Institute NSW CD Fellow
Group Leader, Gynaecology Oncology Group
LS236, University Drive
University of Newcastle
Callaghan NSW 2308 Australia
Email: pradeep.tanwar@newcastle.edu.au
Phone: +61-2-49215148

Disclosure statement: The authors have nothing to disclose

Table S1. List of Primers used for genotyping

Transgene	Forward Primer	Reverse Primer
TCF/Lef:H2B/GFP	5'ACAACAAGCGCTCGACCAT CAC3'	5'AGTCGATGCCCTTCAGCT CGAT3'
Ddx4cre	5'CACGTGCAGCCGTTTAAGCC GCGT3'	5'TTCCCATTCTAAACAACA CCCTGAA3'
Ctnnb1^{tm1Mmt}	5'GACACCGCTGCGTGGACAA TGA3'	5'GTGGCTGACAGCAGCTTT TCTA3'
ROSA26$^{flGFP\text{-}NLS\text{-}lacZ}$	5'AAA GTC GCT CTG AGT TGT TAT3'	5'TCC AGT TCA ACA TCA GCC GCT ACA3'

Table S2. Antibody table and dilutions/conditions/provider with catalogue number

Antibody	Provider	Catalogue No	Dilution
AMH/MIS	Santa Cruz Biotechnology	sc-6886	1:100
βcatenin	BD Transduction Labs	610154	1:200
Ecadherin	Cell Signalling Technology	#3195	1:200
GFP	Abcam	ab6556	1:2500
Inhibinα	Biogenex	AM446-5M	Ready to use
GCNA1	Dr George Enders		1:50
βgal	Abcam	ab9361	1:200

SFigure 1. Ctnnb1$^{ex3/lacZ}$cko mice showed lacz expression in ovarian germ cells. LacZ expression was only observed in oocytes of mutant ovaries indicating germ cell specific cre activity (A-P). Gross images of control and mutant ovaries after β-galactosidase staining (A-H). Arrowheads mark lacZ positive oocytes. No lacZ staining in ovaries from control animals (E-H and M-P). Ovarian sections in panel I-P were counterstained with nuclear fast red stain. Bars:100μm, if not specified in a panel.

SFigure 2. Ctnnb1$^{ex3/lacZ}$cko mice showed enhanced βcatenin expression in lacz-positive cells. Increased βcatenin (green) expression was observed in lacZ-positive cells of mutant ovaries (B&D). No lacZ expression was observed in control ovaries (A&C). lacZ expression (red) was observed only in GCNA-positive cells (green) of ovaries collected from mutant mice (F&H). No lacZ expression was observed in GCNA-positive cells of control ovaries (E&G). Nuclei are marked by DAPI. Bars:100μm.

SFigure 3. Overactive Wnt signalling leads to defective embryonic development in mutant mice. 10.5 dpc pregnant uteri from mutant females showing reduced growth (B) as compared to control females (A). No significant difference was observed in the number of implantation sites among control and mutant mice (N=3/each; C). A representative gross image of control gravid uteri showing enhanced vasculature at an implantation site (arrowheads; D). Uteri from mutant mice (N=3) did not show a similar increase in vasculature at implantation sites (arrowhead; E-F). H&E stained sections of implantation sites from control and mutant mice (G and H). A significant reduction in weight of implantation sites in mutant mice compared to controls (I). Panel J showing a representative image of a normal 10.5dpc embryo isolated from a control mouse. Due to abnormal

development, mutant 10.5dpc embryos could not be isolated. Panel D and E is high magnification image of boxed area in panel A and B, respectively. Bars: 1cm (A and B) and 100μm (D, E, G, H and J).

SFigure 4. Sustained Wnt/βcatenin signalling leads to abnormal embryonic development at 13.5dpc. A gross image of 13.5 dpc pregnant uteri from a mutant mouse (A and B). Comparison of uterine size collected from a 13.5 dpc pregnant mutant mouse and a 10.5 dpc control mouse (C). Bars: 1cm (A) and 100μm (B and C).

SFigure 5. Constitutive activation of Wnt/βcatenin signalling in 10.5dpc Ctnnb1$^{ex3/lacZ}$cko embryos. 10.5 dpc embryos collected from control females showed mainly a membranous expression of βcatenin (A-C). Cytoplasmic and nuclear accumulation of βcatenin in mutant embryos (D-I). Nuclei are marked by DAPI. Bars: 100μm.

SFigure 6. Localization of Ecadherin in control and mutant 10.5dpc embryos. Increased expression of Ecadherin in mutant embryos (D-I) compared to controls (A-C). Nuclei are marked by DAPI. Bars: 100μm.

SFig. 1

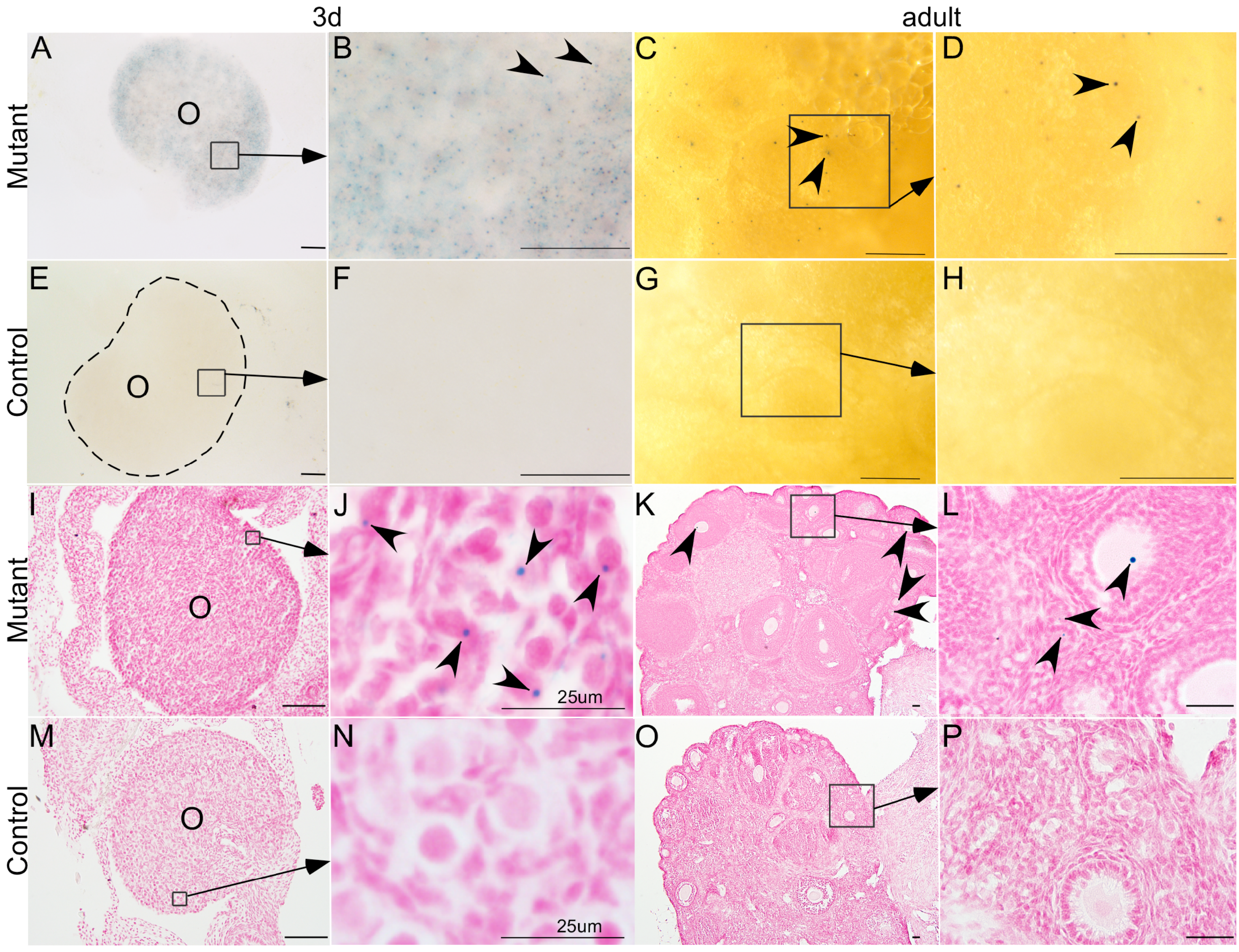

3d
adult
A
B
C
D
Mutant
O
E
F
G
H
Control
O
I
J
K
L
Mutant
O
25um
M
N
O
P
Control
O
25um

SFig. 2

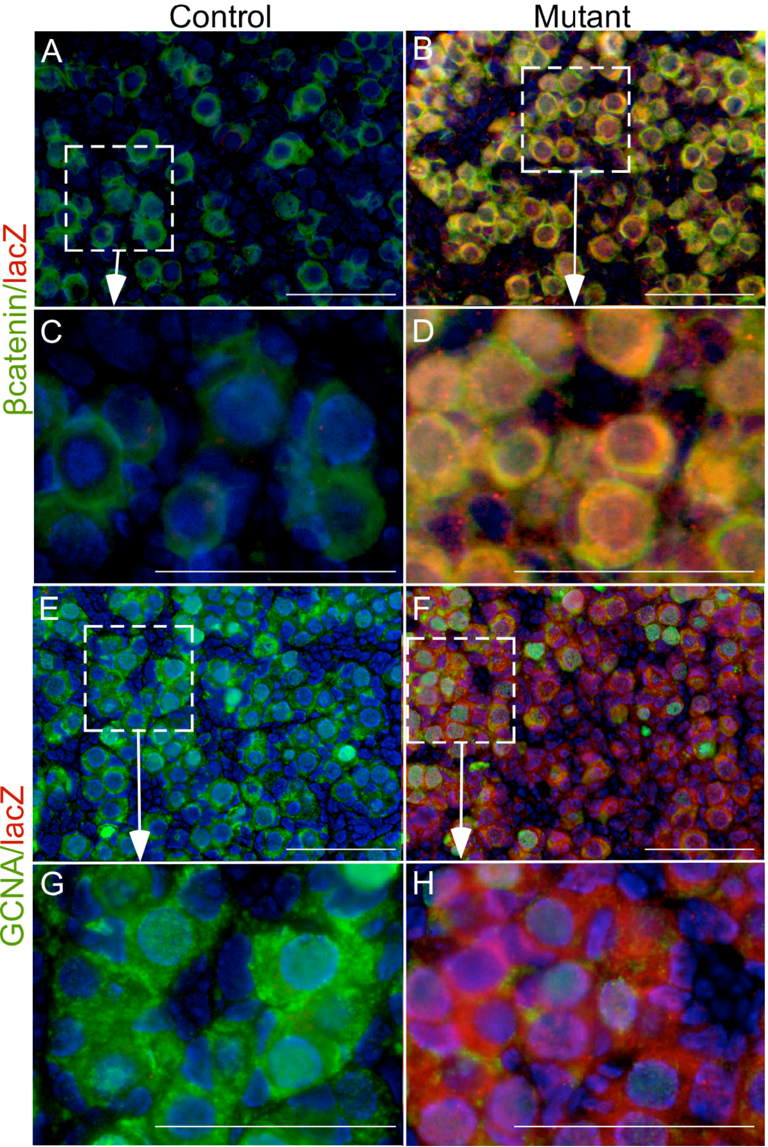

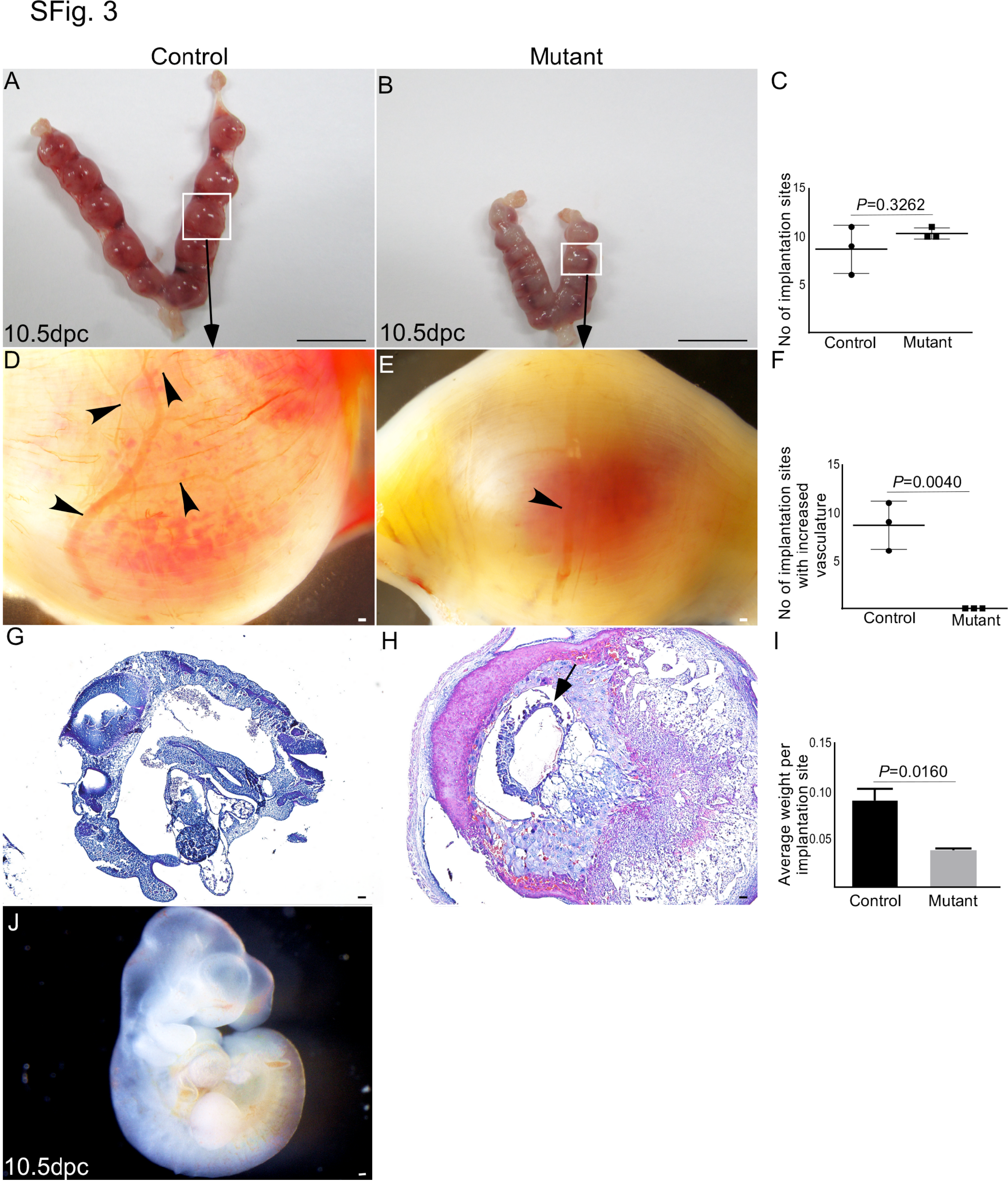
SFig. 3
Control
Mutant
A
10.5dpc
B
10.5dpc
C
No of implantation sites
P=0.3262
Control
Mutant
D
E
F
No of implantation sites with increased vasculature
P=0.0040
Control
Mutant
G
H
I
Average weight per implantation site
P=0.0160
Control
Mutant
J
10.5dpc

SFig. 4

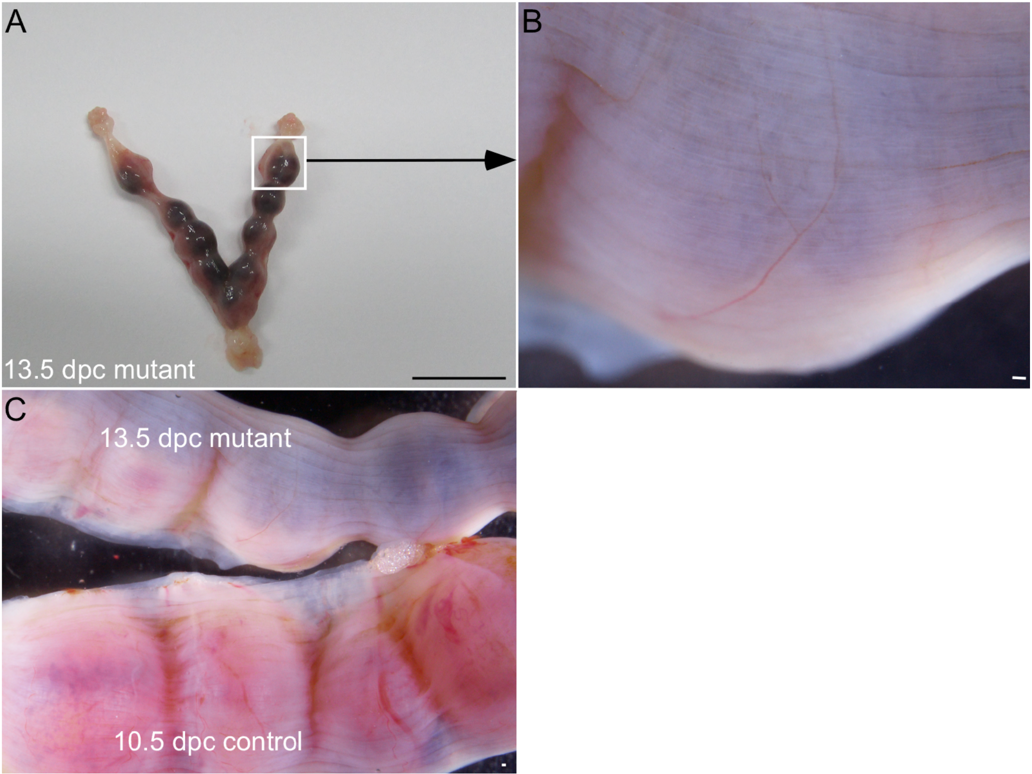

SFig. 5

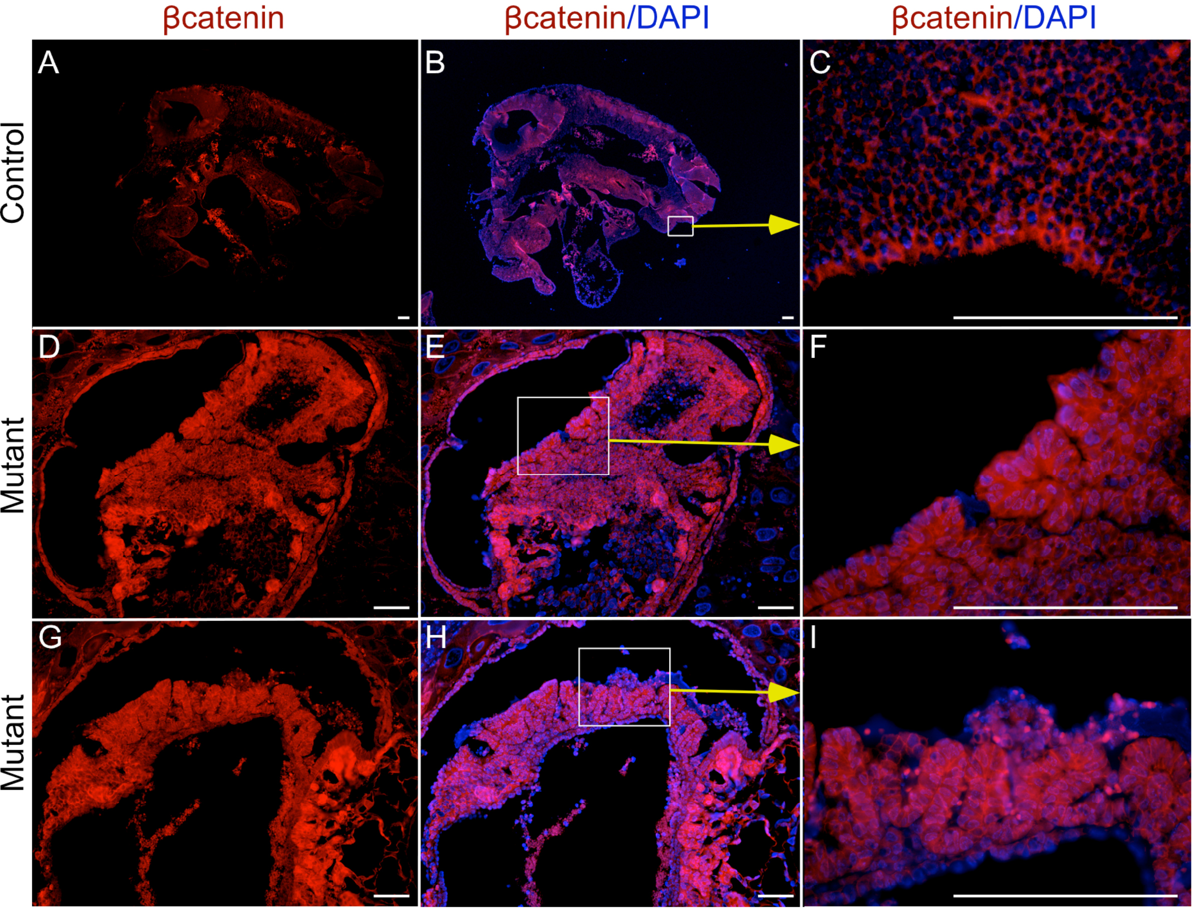

βcatenin
βcatenin/DAPI
βcatenin/DAPI
Control
Mutant
Mutant
A
B
C
D
E
F
G
H
I

SFig. 6

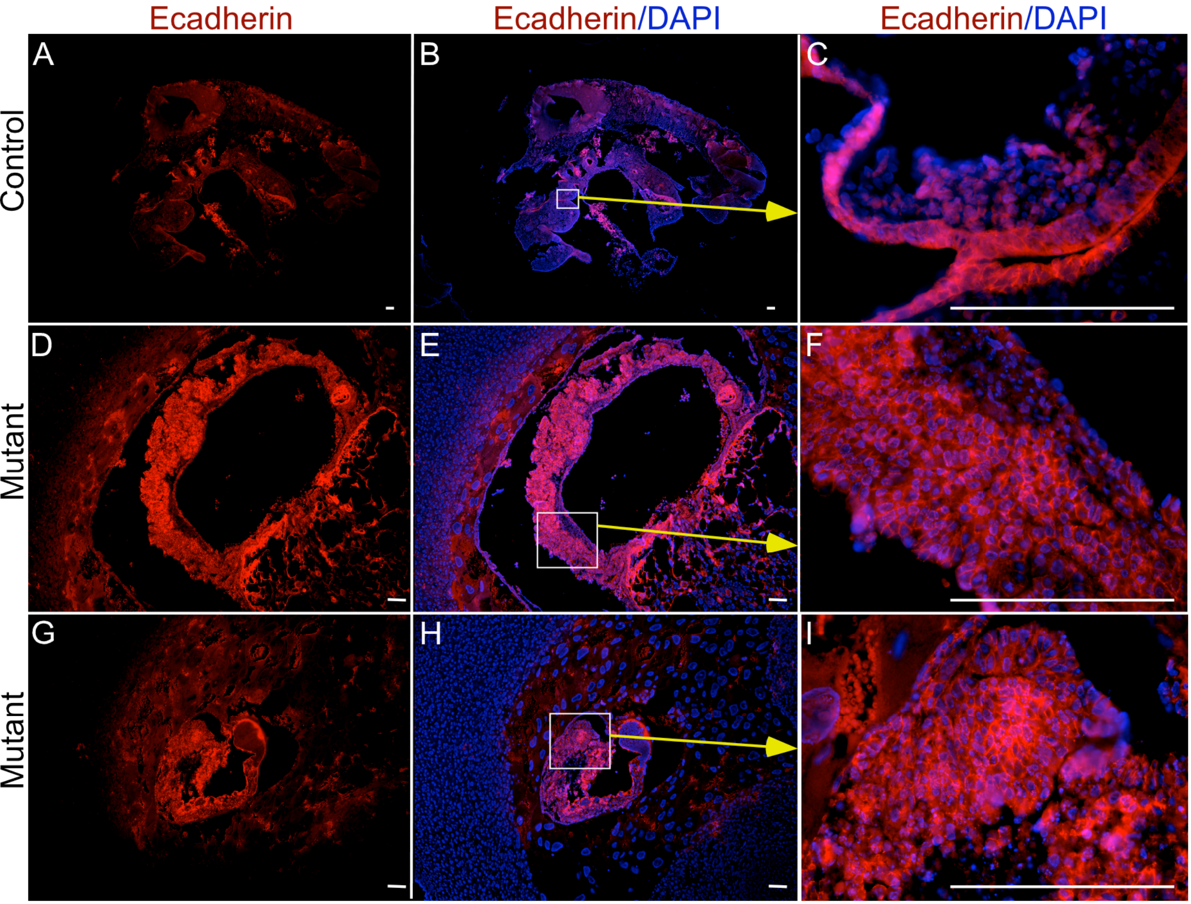
Ecadherin
Ecadherin/DAPI
Ecadherin/DAPI
Control
Mutant
Mutant
A
B
C
D
E
F
G
H
I
